# Prevalence of *Leishmania* species among patients with cutaneous leishmaniasis in Qassim province of Saudi Arabia

**DOI:** 10.1186/s12889-019-6710-8

**Published:** 2019-04-05

**Authors:** Zafar Rasheed, Ahmed A. Ahmed, Tarek Salem, Mohammed S. Al-Dhubaibi, Ahmad A. Al Robaee, Abdullateef A. Alzolibani

**Affiliations:** 1 Department of Medical Biochemistry, College of Medicine, Qassim University, P.O. Box 6655, Buraidah, 51452 Saudi Arabia; 2 Research Center, College of Medicine, Qassim University, Buraidah, Saudi Arabia; 3 Department of Dermatology, College of Medicine, Qassim University, Buraidah, Saudi Arabia

**Keywords:** Cutaneous leishmaniasis, *L. major*, *L.tropica*, *L. infantum/donovani*, Qassim, Saudi Arabia

## Abstract

**Background:**

Leishmaniasis is a parasitic infection endemic in more than ninety countries of the world. The cutaneous leishmaniasis (CL) is a most common form of leishmaniasis and it remains to be a major public health issue in Saudi Arabia. This study was undertaken to investigate the *Leishmania* species responsible for CL infection in different provinces of Qassim, Saudi Arabia.

**Methods:**

Skin biopsies were obtained from CL patients and DNA was extracted using the Magna pure system. *Leishmania* species were identified by highly specific/sensitive quantitative and qualitative PCR.

**Results:**

Out of total 206 CL biopsies, 49.5% biopsies were found to be positive for *Leishmania major* (*L. major*), 28.6% biopsies were positive for *Leishmania tropica (L. tropica)*, 3.9% were found to be positive for *Leishmania infantum/donovani (L. infantum/donovani)*. Not only have these, all tested CL biopsies showed negative test for *Leishmania mexicana (L. mexicana)* and *Leishmania viannia* (*L. viannia*).

**Conclusions:**

This is the first comprehensive study that shows the majority of CL in Qassim was caused by *L. major* and *L. tropica*. To the best of our knowledge, this is the very first report that shows the occurrence of *L. infantum/donovani* in Saudi Arabia. This requires higher alert to the Ministry of Health of Saudi Arabia to take proactive actions in preventing the onset of *L. major*, *L. tropica, L. infantum* and *L. donovani* infections.

## Background

Leishmaniasis is a parasitic infection endemic in more than ninety countries of the world and the cutaneous leishmaniasis (CL) is a most common form of this infection caused by phlebotomine sand fly [1]. The World Health Organization reported in 2016 that about 15 million individuals have leishmaniasis and more than 360 million individuals are breathing in those regions which are prone for this infection and this infection causes ~ 70,000 deaths per year [1, 2]. It is now well documented that CL is caused by more than 22 different species of the genus *Leishmania* (*L*) but their prevalence varies from region to region [3, 4]. Identification of specific *L.* species is important for the prescription of appropriate therapy [5]. Treatment of CL patients without identification of *L.* species cause harmful effects to the patients as the attribution of the relative importance of specific *L.* species in humans are reported [6]. In general, diagnosis is still based on clinical symptoms, microscopic parasitic detection and tissue culturing of promastigotes. However in cases with promastigotes culture, additional efforts should also be needed such as biochemical and serological analysis for further characterization of parasites [7]. These additional efforts for the determination of *L.* species are time taken and are not sensitive, not accurate and sometime give wrong information [8]. Recently, several PCR based detection of *L.* species were developed, which are rapid, sensitive and accurate and now become a powerful approach to determine the *L.* species types at all levels of detection such as genus, complex, and species [9, 10]. Recently, Abuzaid et al. have extensively reviewed the prevalence of CL in Saudi Arabia. They noticed that CL remains an unsolved public health issue of the country [10]. Although, CL is endemic in all over the regions of Saudi Arabia but the majority of patients are continuously reported in Riyadh, Hassa, Aseer, Hail, Madinah, Taif and also in Qassim [10–13]. Despite of all taking care by the Ministry of Health, Saudi Arabia but CL remains to be a major health issue of the country, which may be due to urbanization and huge population immigration [10]. As the information on the diversity of *L.* species in different provinces of Saudi Arabia is poor, therefore the current study was aimed to identify the *L.* species in different provinces of Qassim, Saudi Arabia using highly specific and sensitive PCR-based approach.

## Methods

### Patients recruitment, biopsies collection and DNA extraction

The study was carried out in accordance with the Code of Ethics of the World Medical Association (Declaration of Helsinki as revised in Tokyo 2004) for humans and the study protocol was approved by the National Plan for Science, Technology and Innovation of Saudi Arabia (NSTIP/KACST # 11-MED1068–09). With the Institutional Review Board (IRB) approval, study subjects were recruited through the dermatology outpatient clinics of Qassim region of Saudi Arabia. Patients were diagnosed after careful clinical examination based on clinical presentation and microscopy and were classified as cutaneous leishmaniasis as described previously [14]. Demographic details of the studied subjects are shown in Table 1. A total of 206 biopsies were taken from cutaneous lesions and DNA samples were extracted from all biopsies by MagNaA pure DNA extraction Pure LC DNA Isolation Kit (cat. # 03186229001, Roche Applied Science, Mannheim, Germany) using MagNA Pure LC Automated Instrument according to the manufacturer’s instruction (Roche) as described previously [15, 16]. The absorbance of DNA solution was monitored at 260 nm and 280 nm to ascertain its concentration and purity by using Perkin Elmer UV-Spectrophotometer (L7110223-D, Lambda XLS, Germany) as described previously [17, 18].

**Table 1:** Demographic and clinic characterization of studied cutaneous leishmaniasis patients in Qassim, Saudi Arabia

SNo.	Parameters	Results
1.	Total number patients	206
2.	Patients age (mean ± SD., years)	34.20 ± 13.3
3.	Gender (Males/Females)	114 Males/92 Females
4.	No. of lesions	1–14
5.	Sites of lesions	Limb, facial or skill
6.	Disease duration (mean ± SD., months)	58.99 ± 43.86

### Real time PCR

Quantitative real-time PCR amplification was carried out for the detection of *L. major*, *L. viannia*, *L. mexicana* and *L. infantum/donovani* by the Light cycler PCR system (Roche Diagnostics, Mannheim, Germany). By the use of CLUSTAL program in Lasergene software (Madison, WI), the amplicons were aligned and nucleotide differences among the various *Leishmania* complexes were determined as described previously [19]. Using primer express software (PE Biosystems, Foster City, CA), appropriate probes and flanking primers were designed to specifically identify each *Leishmania* complex. The following primers/probe combination for each complex was designed: *L. major* complex: F-5’TTCTGCTCCGTCGGTGTAGA3’, R-5’GCTTTCGATTGGCTACGACAA3’; and Lmaj-probe 5′-CCTGTCAGGAATTCCACAAA-3′; *L. infantum/donovani* complex: F-5’CCAGATGCCGACCAAAGC3’, R-5’CGCGCACGTGATGGATAAC3’ and Lid-probe 5′-ATCGGCAGGTTCT-3′; *L. mexicana* complex: F-5’CCAGTCCCAGAACACAAACATG3’, R-5’CCTATCGACCAACACAGAAAAGG3’ and Lm probe 5′-ATGCCGAACTCCCGAA-3′; *L. viannia* complex: F-5’CAACAAAATGCTTCGCAACAG3’, R-5’CGCAACGCCTTCATGGA3’ and LV-probe 5′-CGACGGGATATTGTTTGACTT-3′. PCR amplification with specific primer for *L. species* was performed in the same ways as described previously [19–21] with some modifications. Briefly, typical profile times used were initial step, 95 °C for 3 min, followed by 40 cycles of denaturation at 95 °C for 15 s. and annealing/extension at 60 °C for 30 s, followed by 4.0 °C up to 24 h. The reactions were conducted in a 20 μl and set up in a 96-well optical reaction plate. The reactants mix are containing 1x fast start master mix (Integrated DNA Technologies, USA), 3.0 pg. to 100 ng of DNA template, 1 μM of each primer (Integrated DNA Technologies, USA). The presence of amplified products (a positive result) was determined by melting curves and values were recorded for estimation the *L. species*. All samples were run again with conventional PCR assays to determine *L. tropica* species.

### Qualitative PCR

The qualitative PCR for *L. tropica* was carried out by using the following primers sequence F-5’TCGTCTGATTCAAAGTTCTC3’, R-5’CACACGCGCACACCGCGATC3’ designed from *Leishmania tropica* GTG1 sequence (GenBank: AY826393.1) as described previously [22]. The Go Taq® Green Mix (cat. # M7123, Promega Corporation, WI, USA) was used according the instructions provided by the manufacturer (Promega, WI, USA). Briefly, the reaction mixture (50 μL) contained 1X Go Taq® Green Master Mix, 1 μM of each primers (Integrated DNA Technologies, USA), and 100–300 ng of the extracted DNA. The amplification was performed by MWG Biotech Inc. Primus 96 Thermal Cycler, 220 VAC (Mfr# TC-9600-226 V; Item # EW-93941-05) using an initial heating of 95 °C for 2 min. 40 cycles were applied with three stages: denaturation (95 °C for 30 s), annealing (55 for 30 s) and extension (72 °C for 1 min). The products of PCR were loaded to 2% agarose gel and electrophoresed at 10–15 V, followed by staining with 0.1 μg/mL ethyl bromide. The presence of bands was then detected by MacroVue UV-20 transilluminator (Hoefer, Inc., MA, USA).

### Positive and negative controls samples

Positive control DNA samples were extracted from the cultures of standard WHO strains of *L. major* MHOM/SU/1973/5ASKH, *L. tropica* MHOM/SU/1974/SAF-K27, *L. infantum* MHOM/TN/1980/IPT1, *L. donovani* MHOM/IN/1980/DD8, *L. mexicana* MHOM/BZ/1982/BEL21, *L. (viannia) braziliens* MHOM/BR/1975/M2903. Negative control DNA samples were extracted from the clinical samples of different dermatological disorders.

## Results

**Fig. 1:**
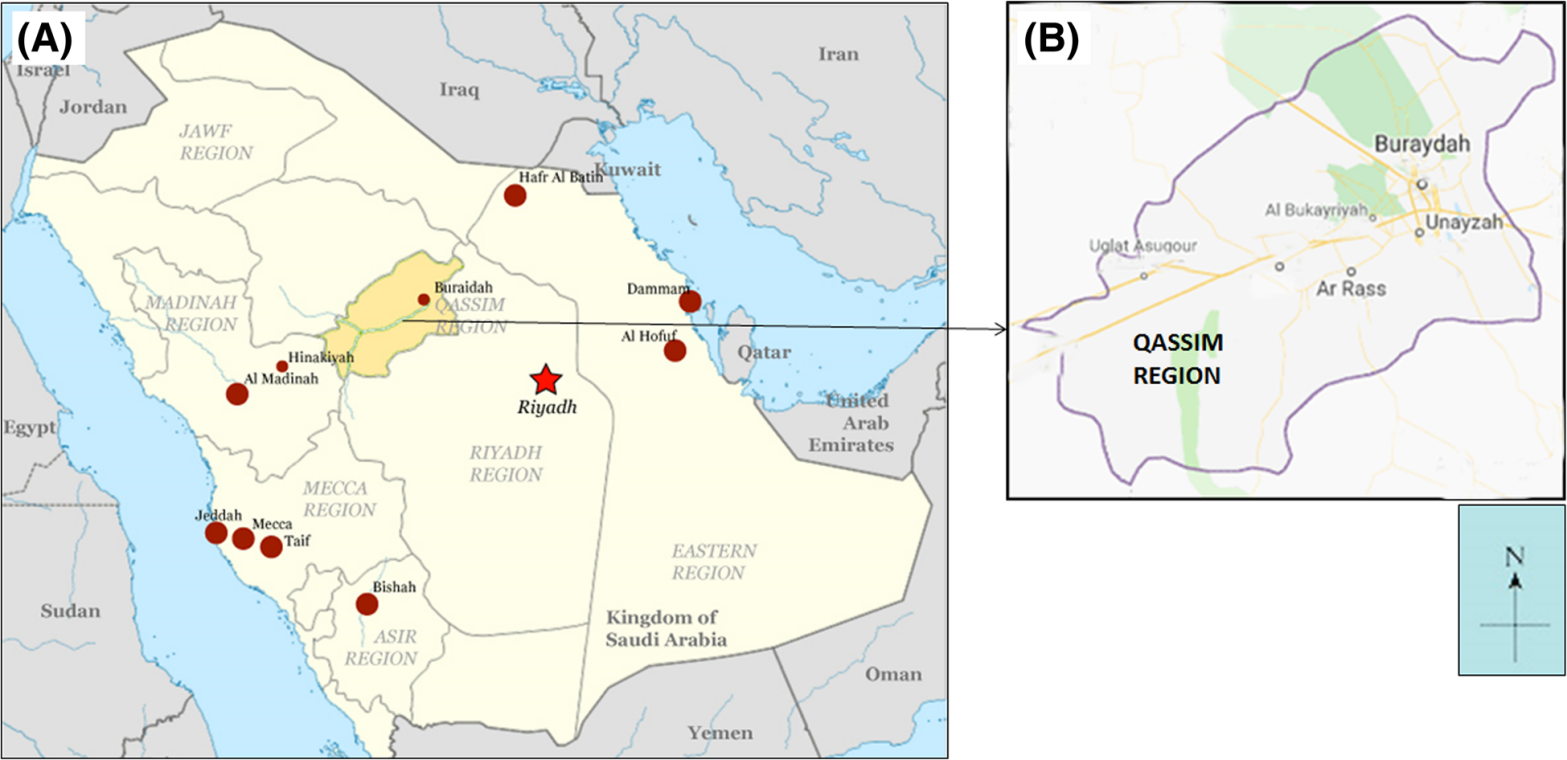
(**a**) Map of Saudi Arabia showing Qassim region in yellow. (**b**) Map of Qassim region of Saudi Arabia showing sites of the study. The maps shown were taken from http://en.wikipedia.org/wiki/File:Saudi_Arabia_location_map.svg

**Fig. 2:**
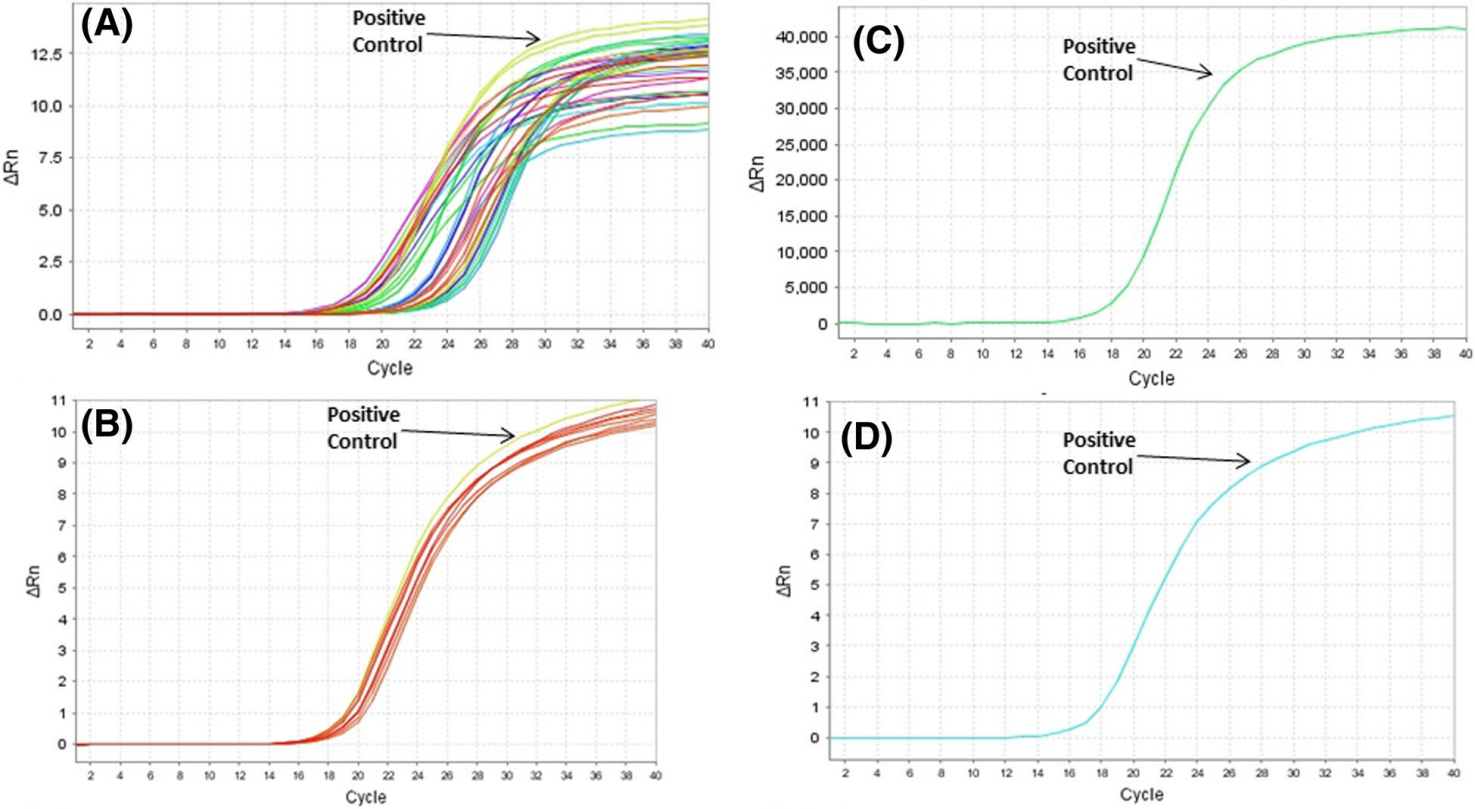
Real time PCR amplification plots of positive samples for (**a**) *Leishmania major* complex, (**b**) *Leishmania infantum/donavani* complex, (**c**) *Leishmania viannia* complex, (**d**) *Leishmania maxicana* complex. Positive control for *L. major* was from strain MHOM/SU/1973/5ASKH, positive control for *L. infantum/donavani complex* was from strain MHOM/TN/1980/IPT1, positive control for *L. mexicana* was from strain MHOM/BZ/1982/BEL21, positive control for *L. viannia* was from *L. braziliens strain* MHOM/BR/1975/M2903. Negative controls and samples negative of specific *L. species* were not detected

**Fig. 3:**
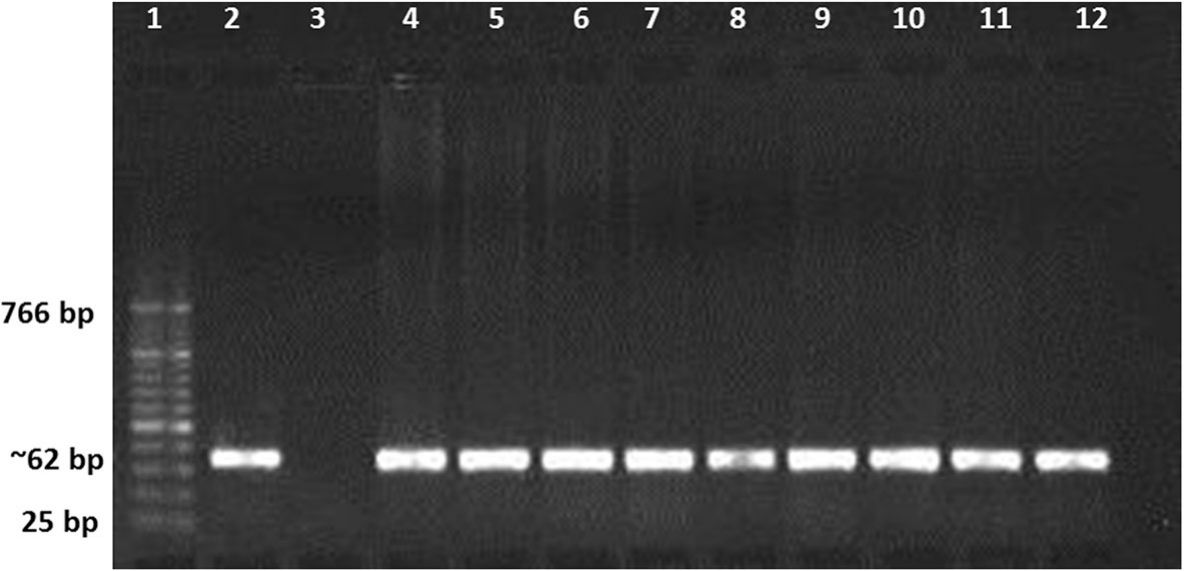
Agarose gel electrophoresis for *Leishmania tropica*. Lane 1: molecular markers (cat. # 474, Biolabs); lane 2: PCR product of *L. tropica* strain MHOM/SU/1974/SAF-K27 (used as positive control); lane 3: PCR product of *L. major* strain MHOM/SU/1973/5ASKH (used as negative control); lane 4: PCR product of β-actin DNA (used as endogenous control); lane 5–12: PCR product from skin biopsies of cutaneous leishmaniasis cases

**Fig. 4:**
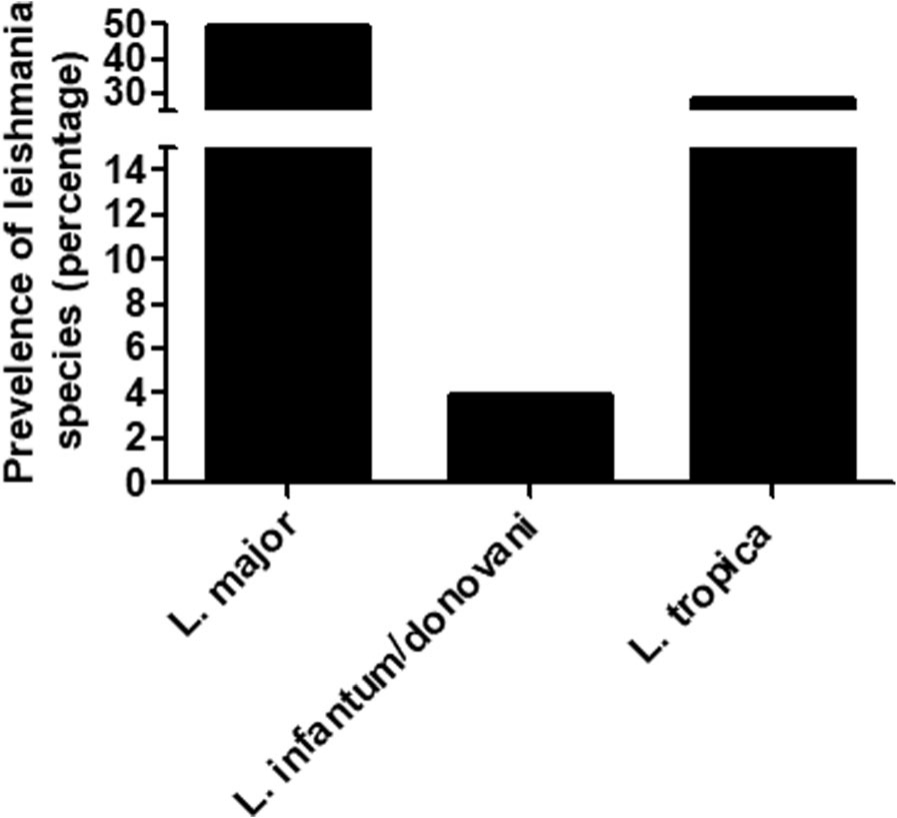
Percent prevalence of *Leishmania* species in patients with cutaneous leishmaniasis

All skin biopsies were collected from cutaneous leishmaniasis (CL) patients, which were attending various dermatological clinics in different provinces of Qassim, Saudi Arabia. Sites of the study are shown in the map of Qassim province of Saudi Arabia (Fig. 1). The majority of the patients were from Buraidah (63%) and Unayzah (19%) and the rest (18%) were Ar Rass, Bukariya, Uglat Asugour, etc. In an attempt to investigate the different types of *L. species* in Qassim region, we used quantitative and qualitative PCR for identification of different *L. species* using specific sets of primers for *L. major, L. infantum/donovani complex, L. mexicana, L. viannia,* and *L. tropica.* Specificity and validity of each quantitative and qualitative PCR assays were tested by the standard WHO. stains. As shown in Table 2, there was a concordance in the characterization of *L. species* by both the quantitative and qualitative PCR assays. Moreover, the results obtained from PCR assays were further validated by using several negative control samples listed in Table 2. Furthermore, no amplification was detected with any of the PCR assays tested for the negative control DNA samples, whereas the presence of extracted DNA was confirmed by β-actin (Table 2). Amplification plots of positive samples for *L. major*, and *L. infantum/donavani* complex are presented in Fig. 2. These amplification plots of positive samples for *L. major*, and *L. infantum/donavani* complex were comparable with their respective positive controls. Importantly, all tested patients samples were found to be negative for *L. viannia and L. maxicana*. Negative controls and samples negative for specific *L. species* were not detected. Whereas, samples positive for *L. tropica* samples were characterized by agarose gel electrophoresis and the details are given in Fig. 3. Specifically, out of total 206 CL biopsies, 102 was found to be positive for *L. major*, 08 was positive for *L. infantum/donovani* complex, and 59 CL biopsies were found to be positive for *L. tropica*. We have also calculated the percent prevalence of these *Leishmania* species among the studied patients. The *L. major* and *L. tropica* are the most abundant types found in 49.5 and 28.6%, respectively, whereas *L. infantum/donovani,* was found in 3.9% of the studied subjects (Fig. 4). The complete data of the studied patients with the prevalence of *L. species* types in Qassim are summarized in Table 3.

**Table 2:** Testing of primers’ sequences for positive and negative controls DNA samples for *leishmaniasis species*

Positive/negative control DNA samples	Source	*L. major* assays	*L. infantum /donovani* assays	*L. mexicana* assays	*L. viannia* assays	*L. tropica* assays	β-actin assays
*L. major* DNA	Culture	Positive	Negative	Negative	Negative	Negative	Positive
*L. tropica* DNA	Culture	Negative	Negative	Negative	Negative	Positive	Positive
*L. infantum* DNA	Culture	Negative	Positive	Negative	Negative	Negative	Positive
*L. donovani* DNA	Culture	Negative	Positive	Negative	Negative	Negative	Positive
*L. mexicana DNA*	Culture	Negative	Negative	Positive	Negative	Negative	Positive
*L. (V).* braziliens DNA	Culture	Negative	Negative	Negative	Positive	Negative	Positive
Vitiligo DNA	Skin	Negative	Negative	Negative	Negative	Negative	Positive
Psoriasis DNA	Skin	Negative	Negative	Negative	Negative	Negative	Positive
Basal cell carcinoma DNA	Skin	Negative	Negative	Negative	Negative	Negative	Positive
Vitiligo blood DNA	Blood	Negative	Negative	Negative	Negative	Negative	Positive
Psoriasis blood DNA	Blood	Negative	Negative	Negative	Negative	Negative	Positive
Acne DNA	Blood	Negative	Negative	Negative	Negative	Negative	Positive
Atopic dermatitis DNA	Blood	Negative	Negative	Negative	Negative	Negative	Positive
SLE DNA	Blood	Negative	Negative	Negative	Negative	Negative	Positive
Normal DNA	Blood	Negative	Negative	Negative	Negative	Negative	Positive

**Table 3:** Prevalence of different leishmaniasis types in Qassim region of Saudi Arabia

SNo.	Parameters	Results
1	Leishmaniasis patients	
1.1	Total number skin biopsies tested	206
1.2	Patients age (mean ± SD., Years)	34.20
1.3	Sex (Males/Females)	194 Males/12 Females
1.4	Disease duration (mean ± SD.,Months)	58.99 ± 43.86
SNo.	Types of leishmaniasis	Results
2	L. major complex	
2.1	Number of biopsies found	102
2.2	Patients age (mean ± SD., Years)	37.61 ± 14.7
2.3	Sex (Males/Females)	92 Males/10 Females
2.4	Disease duration (mean ± SD., Months)	60.8 ± 42.5
3	L. infantum/donovani complex	
3.1	Number of biopsies found	08
3.2	Patients age (mean ± SD., Years)	30.2 ± 5.6
3.3	Sex (Males/Females)	08 Males
3.4	Disease duration (mean ± SD., Months)	67.5 ± 13.9
4	L. tropica complex	
4.1	Number of biopsies found	59
4.2	Patients age (mean ± SD., Years)	30.90 ± 7.613
4.3	Sex (Males/Females)	59 Males
4.4	Disease duration (mean ± SD., Months)	63.2 ± 55.6

## Discussion

This is the first comprehensive report that shows the majority CL in central region of Saudi Arabia was caused by *L. major* and *L. tropica*. Apply the PCR-based identification of specific *L. species*, this report also demonstrated the occurrence of *L. infantum/donovani* for the first time in Saudi Arabia. Based on the endemic nature of CL in gulf countries including Saudi Arabia and also unidentified *L. species* [10, 23], we have selected five *Leishmania* species namely *L. major*, *L. infantum/donovani* complex, *L. mexicana*, *L. viannia*, and *L. tropica* for the detection of CL infection. It is now well established that the PCR based identification of leishmaniasis infection has now been proved to be a better method as compared with other diagnostic methods in terms of specificity and accuracy [8, 9, 24, 25]. Quantitative real time PCR is an excellent diagnostic way which uses fluorescent labeling for the continuous watching of amplicon generation throughout the amplification process [8, 9, 26]. The most important advantages of quantitative real time PCR are that it is highly sensitive, accurate and extremely faster as compared to other available techniques for the detection of a target *Leishmania* species [8, 9, 19, 26]. Studies have shown that diagnosis of *Leishmania* species through quantitative real time PCR is 78 and 76% more sensitive than culture and expert microscopy, respectively [19, 24–26]. Therefore, we used quantitative real time PCR for all studied *L. species* except for *L. tropica.* In case of *L. tropica* and *L. major,* similar repeats in their sequences were reported at their flanking sequences and microsatellite, while designing of the PCR primer pairs. Furthermore, the previous studies also revealed that the microsatellites repeats were likely to be located on at least eight different chromosomes [22, 27]. For these reasons, the analysis of *L. tropica* was done through qualitative PCR analysis by using the well-designed GTG1 sequence (Gene bank: AY826393.1), which was well established for *L. tropica* only [22]. During the testing of all designed primers for PCR assays on positive and negative standard DNA samples, our results also showed that the primers on GTG1 sequence showed positive results only for *L. tropica*, whereas other tested *L. species* including *L. major* showed negative results.

In this study, CL infected skin biopsies were used from those CL patients which were attending various dermatological clinics in different provinces of Qassim, Saudi Arabia. The majority of the patients for this study were from Buraidah (63%) and Unaizah (19%) and the rest (18%) were from other parts of Qassim region including Ar Rass, Bukariya, Uglat Asugour (Fig. 1). All patients were diagnosed as CL based on clinical presentation and microscopy as described previously [14]. Our high sensitive PCR based analysis shows the *L. major* and *L. tropica* are the most abundant types of *L.* species found in 49.5 and 28.6% of CL patients, respectively. In support of our results, previous studies on parasite identification proved that *L. major* and *L. tropica* are the main causative CL species in Saudi Arabia [8, 10, 28, 29]. *L. major* has also been identified as the main *Leishmania* species in Riyadh and Al-Hassa regions [10] and now appears to be randomly transmitted in all over Saudi Arabia including Qassim [10, 28–30]. The characterization of isoenzyme *L. major* zymodeme LON-4 has been identified that *L. major* is a typical parasite present in all over Saudi Arabia [10]. Whereas, *L. tropica* has been found to be a main *L. species* responsible for causing CL infection in Aseer and Al-Bahah provinces which are in southwest part of Saudi Arabia [10, 28, 29]. In view of these and together with the occurrence of *Phlebotomus papatasi*, a *L. major* vector in many provinces of the country [10], it is now well established that *L. major* is responsible for causing CL infection in most of the provinces of the country, while *L. tropica* is only convicted in the southwestern zone [2]. However, recently it was demonstrated that *L. tropica* is also dispersed in other parts of the country other than southwestern area [10, 28–30]. As CL is well known to be an endemic disease not only in Saudi Arabia but also in other gulf countries and the main causative species were repeated found to be *L. major* and *L. tropica* in these countries [10, 23]. Addition of this study in different provinces of Qassim region has now been confirmed that the main causative *Lieshmania* species of cutaneous leishmaniasis in this region are *L. major* and *L. tropica*.

Anthroponotic transmission is not only the characteristic of *L. tropica*, but also for *L. donovani* complex, which is usually found in the Indian subcontinent including India, Bangladesh, Nepal, Sudan, Ethiopia, Kenya and Somalia [3, 31]. It is also reported that *L. donovani* is the cause of both anthroponotic and zoonotic characteristics in East Africa [3, 32]. On the other hand, *L. infantum* occurs in the Mediterranean, the Middle East, Afghanistan, Iran, Pakistan and Brazil, although sporadic cases have been reported in Central Asia, China, Mexico and Central and Latin America [31, 33]. Notably, immunosuppressed adults and children are at higher risk of clinical disorder due to *L. infantum* than immunocompetent adults [34]. Transmission of *L. infantum* infection is considered predominantly zoonotic, with domestic animals being the major reservoir [33, 35].

To the best of our knowledge, this is the first report that shows the occurrence of *L. infantum/donovani* complex in Saudi Arabia. Our data showed that 3.9% of studied leishmaniasis samples were found to be positive for *L. infantum/donovani* complex. The lower rate of occurrence of *L. infantum/donovani* complex in the studied region, suggested that this infection might be from outside of Saudi Arabia due to the huge population movement in the country especially from India, Bangladesh, Nepal, Sudan, Ethiopia, Kenya, Somalia, Afghanistan and Pakistan, where *L. infantum/donovani* complex is widely spread [3, 31, 32]. Not only have these, we have also tested the occurrence of *L. mexicana* and *L. viannia* in the studied patients samples, our test showed negative results for these *L. species*, strongly suggesting that the Qassim, the central region of Saudi Arabia is free of *L. mexicana* and *L. viannia* infection.

## Conclusions

Cutaneous leishmaniasis is now considered to be a major dermatologic problem in Saudi Arabia with particular insight in the reported provinces. Using highly specific and sensitive PCR-based identification, this study determined that the majority cutaneous leishmaniasis patients in Qassim province of Saudi Arabia were caused by *L. major* and *L. tropica*. However, few leishmaniasis cases were found to be positive for *L. infantum/donovani* complex. This requires higher alert to the Ministry of Health of Saudi Arabia to take proactive actions in preventing the onset of *L. major*, *L. tropica* and *L. infantum/donovani* infections.

## Abbreviations

CL: Cutaneous leishmaniasis
KACST: King Abdulaziz City for Science and Technology
L. infantum/donovani: Leishmania infantum/donovani
*L. major*: Leishmania major
*L. mexicana*: Leishmania mexicana
*L. tropica*: Leishmania tropica
L. viannia: Leishmania viannia
NSTIP: National Plan for Science, Technology and Innovation
